# Feasibility of implementing extracorporeal cardiopulmonary resuscitation in a middle-income country: systematic review and cardiac arrest case series

**DOI:** 10.62675/2965-2774.20250320

**Published:** 2025-05-06

**Authors:** Gabriel Afonso Dutra Kreling, Pedro Vitale Mendes, Luis Carlos Maia Cardozo, Karina Turaça Kasahaya, Marcelo Park, Ludhmila Abrahão Hajjar, Ian Ward A. Maia

**Affiliations:** 1 Hospital das Clínicas Faculdade de Medicina Universidade de São Paulo São Paulo SP Brazil Intensive Care Unit, Emergency Department, Hospital das Clínicas, Faculdade de Medicina, Universidade de São Paulo - São Paulo (SP), Brazil.; 2 Hospital das Clínicas Faculdade de Medicina Universidade de São Paulo São Paulo SP Brazil Emergency Department, Hospital das Clínicas, Faculdade de Medicina, Universidade de São Paulo - São Paulo (SP), Brazil.

**Keywords:** Extracorporeal membrane oxygenation, Heart arrest, Emergency medical system, Critical care medicine

## Abstract

**Objective:**

To evaluate the consistency of current evidence supporting the use of extracorporeal cardiopulmonary resuscitation to treat patients with cardiac arrest and assess the plausibility of implementing an extracorporeal cardiopulmonary resuscitation program in a public health care system hospital in a middle-income country.

**Methods:**

A systematic review, meta-analysis, meta-regression analysis, and trial sequence analysis were performed to assess the consistency of current evidence supporting the use of extracorporeal cardiopulmonary resuscitation to treat patients with cardiac arrest. Additionally, a local cardiac arrest registry was analyzed to identify potential patients eligible for extracorporeal cardiopulmonary resuscitation.

**Results:**

The systematic review included 31 studies. The main and sensitivity analyses consistently demonstrated that extracorporeal cardiopulmonary resuscitation was associated with favorable neurological outcomes (cerebral performance category 1 or 2, RR 1.45, 95%CI 1.19 - 1.77) and survival (RR 1.29, 95%CI 1.10 - 1.52). Age was inversely related to neurological outcome and survival. Our cardiac arrest registry included 55 patients with a median age of 54 years and a survival rate of 18.2% (10/55). Survivors had an initial shockable rhythm. In the most inclusive scenario, 13 patients would have been eligible for extracorporeal cardiopulmonary resuscitation. Under stricter criteria (age ≤ 65 years, low-flow time ≤ 30 min, and number of defibrillations ≥ 3), 4 patients would have been eligible.

**Conclusion:**

Extracorporeal cardiopulmonary resuscitation in patients with refractory cardiac arrest is associated with improved neurological outcomes and survival. The use of an extracorporeal cardiopulmonary resuscitation program in our hospital is plausible. Using conservative eligibility criteria, we estimate that at least four patients would be eligible for extracorporeal cardiopulmonary resuscitation within six months of the program initiation.

## INTRODUCTION

Extracorporeal cardiopulmonary resuscitation (ECPR) has been increasingly explored as an adjunct to conventional cardiopulmonary resuscitation (CCPR) in the management of cardiac arrest.^(1,2)^ Despite advances in CCPR, outcomes for out-of-hospital cardiac arrest (OHCA) events remain poor, with a 1-y survival of 8.8%, often accompanied by unfavorable neurological outcomes.^(3)^ In contrast, ECPR has demonstrated more favorable outcomes in selected patients, with 6-month survival rates ranging from 31.5 to 40% and minimal or no neurological impairment.^(1,2)^ Furthermore, the current literature suggests that for patients with OHCA receiving ECPR, the number needed to treat for survival is 11, and the number needed to treat for favorable neurological outcomes is 12.^(4)^

Given the global annual incidence of OHCA, which is estimated to range from 30.0 to 100.2 events per 100,000 people, ECPR could have a significant impact on survival and neurological outcomes.^(5)^ However, concerns about the cost-effectiveness of ECPR remain, as costs are influenced by factors such as health care setting, duration of support, and patient outcomes. While ECPR has been shown to be cost effective for select patients with refractory OHCA in high-income countries, data on ECPR cost effectiveness in middle-income countries are scarce.^(6)^ Understanding these factors is crucial for assessing the potential benefits and challenges of implementing ECPR in resource-constrained environments.

In Brazil, the public health system does not currently fund the use of extracorporeal membrane oxygenation (ECMO) for respiratory or cardiovascular support.^(7)^ Despite this, our hospital has independently funded ECMO for selected patients since 2011, achieving outcomes similar to those reported in the international literature.^(8-12)^

Therefore, we hypothesized that establishing a structured ECPR program is potentially plausible and cost-effective in a public health system of a metropolitan area in Brazil. To test this hypothesis, we proposed the following steps: (1) assess the consistency of the current evidence supporting the use of ECPR to treat patients with cardiac arrest through a systematic review and meta-analysis; (2) analyze the frequency of patients with cardiac arrest treated at our emergency department to identify potential candidates; (3) define local eligibility criteria for ECPR; (4) train a multidisciplinary team; (5) start implementing ECPR support; (6) evaluate the program plausibility and cost effectiveness after implementing ECPR; and (7) reanalyze the program continuity. In this study, we aimed to describe the initial steps in establishing our ECPR program, focusing on the first three phases of the outlined plan.

Therefore, to analyze the first three steps cited: 1) We conducted a systematic review and meta-analysis with trial sequential analysis and meta-regression to update and test the consistency and heterogeneity of the current evidence supporting the use of ECPR to treat patients with cardiac arrest; 2) Using a local cardiac arrest registry, we sought to identify eligible candidates for ECPR and determine their frequency; and 3) Combining our findings on the consistency and heterogeneity of the current evidence supporting the use of ECPR to treat patients with cardiac arrest with data from our cardiac arrest registry, we aimed to define local criteria for ECPR inclusion.

## METHODS

We conducted a systematic review and meta-analysis following the Preferred Reporting Items for Systematic Reviews and Meta-Analyses (PRISMA) guidelines.^(13,14)^ The study protocol was registered on the International Platform of Registered Systematic Review and Meta-analysis Protocols (INPLASY) under registration number 202350011.^(15)^ The cardiac arrest registry data were retrieved from a prospectively and consecutively collected emergency department database. This study was approved by the Research Ethics Committee of *Hospital das Clínicas*, *Universidade de São Paulo* (approval number 107.443), and informed consent was waived due to the observational nature of the study.

### Search strategy and selection criteria

We conducted a systematic search of the MEDLINE, Lilacs, ScienceDirect, and Web of Science databases to identify relevant studies comparing the use of ECPR and CCPR to treat patients with cardiac arrest. No publication period limits were applied. A detailed search strategy is provided in the Supplementary Material. Two reviewers (GADK and MP) independently assessed titles and abstracts using predefined criteria. Disagreements were arbitrated by a third author (PVM) using the Rayyan systematic review platform. Full-text eligibility assessment was performed independently by GADK and PVM, with disagreements resolved through discussion and arbitration by MP.

### Eligibility criteria

Studies were included if they met the following criteria:

–Included patients with cardiac arrest
–Included adult patients (≥ 18 years old)
–Included patients supported with ECPR
–Were randomized controlled trials (RCTs) or observational studies with a matched control group
–Reported intrahospital or out-of-hospital cardiac arrest
–Reported shockable or nonshockable initial cardiac rhythm
–Reported any cardiac arrest etiology
–Reported any comorbidity
–Included or did not include target temperature management
–Included any no- or low-flow time

Studies were excluded if they met the following criteria:

–Included children or neonates

The exposure of interest was the use of ECPR as an adjunct to standard care, whereas the control group received standard care alone during cardiac arrest management. Only randomized trials or observational studies with structured matching (propensity score matching or coarsened exact matching) were included in the primary analyses. Logistic regression-adjusted studies were included in the sensitivity analyses to increase the consistency of the findings but were not included in the main analysis because they did not report a clear absolute number of paired events [mortality and number of patients with cerebral performance category (CPC) 1 or 2].

### Outcomes and subgroup analysis

The primary outcome was the last reported favorable neurological outcome, which was defined as a CPC of 1 or 2. The secondary outcome was the last reported survival. To test the consistency of the outcomes, a priori sensitivity (using logistic regression-adjusted studies) and subgroup (meta-regression using age, location of cardiac arrest, and, if possible, low-flow time) analyses were planned to explore potential sources of statistical heterogeneity.

### Data collection and risk of bias assessment

We analyzed the full-text manuscripts of the included studies and summarized their main characteristics. The data were extracted from each study by two authors (MP and PVM) using Excel data extraction. The data extracted are described in the Supplementary Material. The assessment of the risk of bias in each trial and outcome was performed by two independent authors (MP and PVM), and discrepancies were discussed among the investigators. The randomized control trials were analyzed according to the Cochrane Risk of Bias tool, version 2 (RoB-2).^(16)^ Observational studies were planned to be analyzed according to ROBINS-I to assess the risk of bias in the results of nonrandomized studies that compared the health effects of two or more interventions.^(17)^

### Cardiac arrest registry

We analyzed data from cardiac arrest events in patients admitted to the Emergency Department of *Hospital das Clínicas*, São Paulo, Brazil, between 12/09/2022 and 05/02/2023. This is the largest academic hospital in Brazil and serves as a referral center for critically ill patients in São Paulo. The analysis included both patients with intrahospital cardiac arrest (IHCA) and those with OHCA. Follow-up assessments were routinely conducted to evaluate intrahospital survival and CPC scores. Patient characteristics and details of cardiac arrest events were documented by a direct observer during clinical interventions, ensuring an accurate and comprehensive representation of each patient’s clinical course and neurological status post-cardiac arrest. All data were prospectively collected via the REDCap™ platform for patients admitted to the emergency department.^(18)^

### Statistical analysis

For continuous variables, the means and standard deviations were extracted. For the manuscripts that did not present means and standard deviations, we estimated these values from the sample size, median, range, or 25^th^ and 75^th^ percentiles using Wan’s technique.^(19)^ We assessed heterogeneity using the Cochran Q statistic and I^2^. Either p < 0.05 or I^2^ > 50% was considered suggestive of high heterogeneity. We analyzed the data using both fixed-effects (Mantel-Haenszel method) and random-effects (DerSimonian method) models. Publication bias was assessed using a funnel plot. To further explore heterogeneity, we planned a meta-regression analysis using the following variables: location of arrest, age, and low-flow time; however, as the low-flow time was presented in few studies, it was not analyzed.

The analyses were performed using the meta (version 7.0-0) and metafor (version 4.4-0) packages in R open-source software (version 4.3.2).^(20,21)^ The metabin function in the meta package was used to calculate the risk ratio (RR). By default, when a study had 0 events in one of the groups (either experimental or control), the function automatically applied a continuity correction by adding 0.5 to the number of events in the group with 0 events. This correction was applied exclusively to studies with 0 events, as per the default behavior of the function, allowing for an appropriate calculation of the RR.^(22)^

To control the risks for type I and type II errors and to assess the need for additional data, we conducted a trial sequential analysis (TSA) of the matched studies (randomized, propensity score matched, and coarsened exact matched) using the open-source TSA version 0.9.5.10 beta software. We assumed a significance level of 0.05 and a power of 80% and applied O`Brien-Fleming boundaries to the alpha spending procedure.^(23)^

## RESULTS

**Figure 1:**
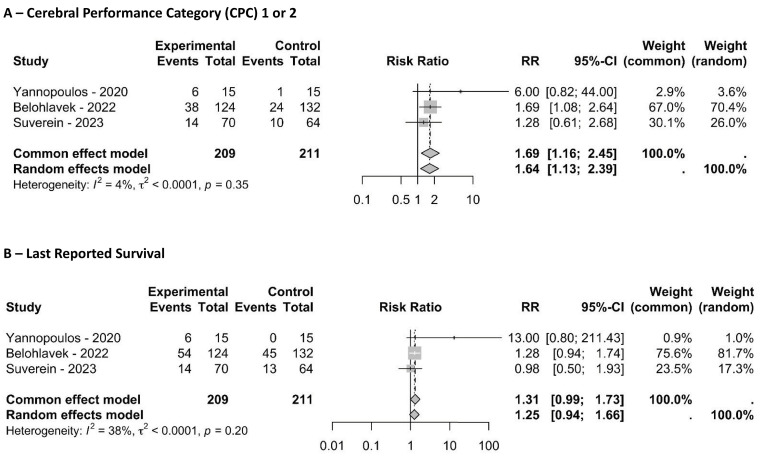
Pooled results from randomized studies. (A) shows the last reported number of patients with favorable neurological outcomes; (B) shows the last reported survival.

**Figure 2:**
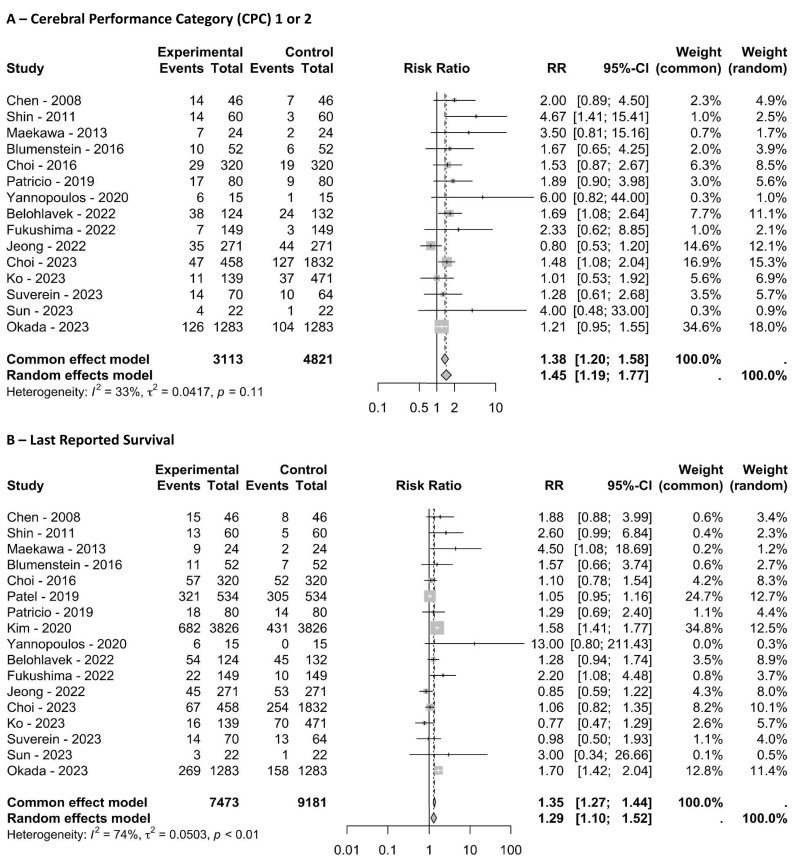
Pooled results from the randomized, propensity score-matched, and coarsened exact-matched studies. (A) shows the last reported number of patients with favorable neurological outcomes; (B) shows the last reported survival.

We identified 31 studies that met our inclusion criteria (Table 1S), including 3 RCTs, 14 propensity score-matched or coarsened exact-matched studies, 6 logistic regression-adjusted studies, and 8 studies that were neither matched nor adjusted. The PRISMA flowchart showing how studies were selected is provided in figures 1S and 2S (Supplementary Material), which illustrates the number of patients studied in each country across randomized and matched studies.

### Randomized controlled trials

**Figure 3:**
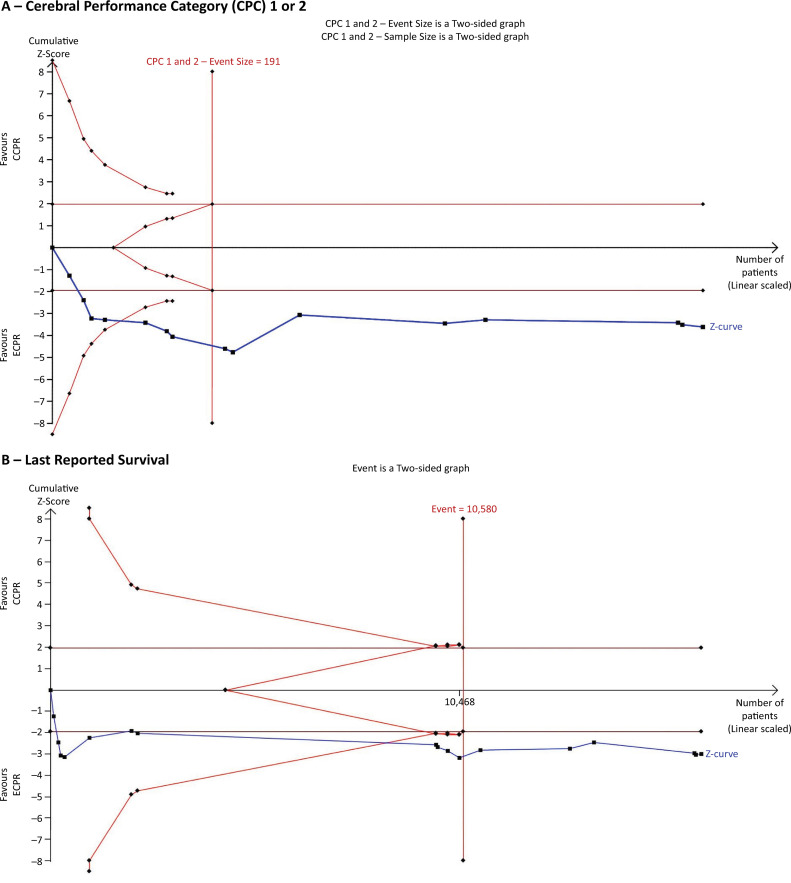
Trial sequential analysis of the cumulative effects of the randomized, propensity score-matched and coarsened exact-matched studies. (A) shows the last reported favorable neurological outcome; (B) shows the last reported survival. CCPR - conventional cardiopulmonary resuscitation; ECPR - extracorporeal cardiopulmonary resuscitation.

In the analysis of the 3 RCTs (Figure 1), the number of patients with the last reported favorable neurological outcome (CPC 1 or 2) was higher in the ECPR group (RR 1.64, 95%CI 1.13 - 2.39; I^2^ = 4%). For survival, the RR was 1.25 (95%CI 0.94 - 1.66; I^2^ = 38%). The risk of bias for these randomized studies is summarized in figure 3S (Supplementary Material).

### Analysis with matched studies

When we included data from the propensity score-matched and coarsened exact-matched studies along with the RCTs (Figure 2), the RR for achieving the last reported favorable neurological outcome was 1.45 (95%CI 1.19 - 1.77; I^2^ = 33%), and the RR for survival was 1.29 (95%CI 1.10 - 1.52; I^2^ = 74%). The risk of bias for these matched studies is presented in table 2S (Supplementary Material).

### Trial sequential analysis

Considering that only randomized studies were included, the total sample from these studies was insufficient to perform a TSA. However, when both randomized studies and observational studies with propensity score matching and coarsened exact matching were incorporated, the cumulative Z curve for the last reported favorable neurological outcome (Figure 3A) crossed both the conventional boundary for benefit and the trial sequential monitoring boundary. Similarly, the Z curve for last reported survival (Figure 3B) crossed both the conventional and trial sequential monitoring boundaries, suggesting that ECPR consistently improved survival and neurological outcomes.

### Heterogeneity and meta-regression

We found no significant effect of the location of cardiac arrest (intrahospital vs. out-of-hospital) on outcomes. For last reported favorable neurological outcome, the meta-regression showed an I^2^ of 24.36%, with an estimated coefficient of -0.68 (95%CI -1.42 - 0.05; p = 0.069) for out-of-hospital cardiac arrests. For last reported survival, we found greater heterogeneity (I^2^ = 73.09%), with an estimated coefficient of -0.54 (95%CI -1.23 - 0.16; p = 0.133), but no significant effect was detected. The meta-regression for age, which included data from randomized and matched studies, revealed an I^2^ of 39.76%, with an estimated coefficient of 0.07 (95%CI 0.01 - 0.13; p = 0.032), suggesting that younger age is associated with better survival outcomes in patients treated with ECPR.

### Study characteristics and publication bias

**Figure 4:**
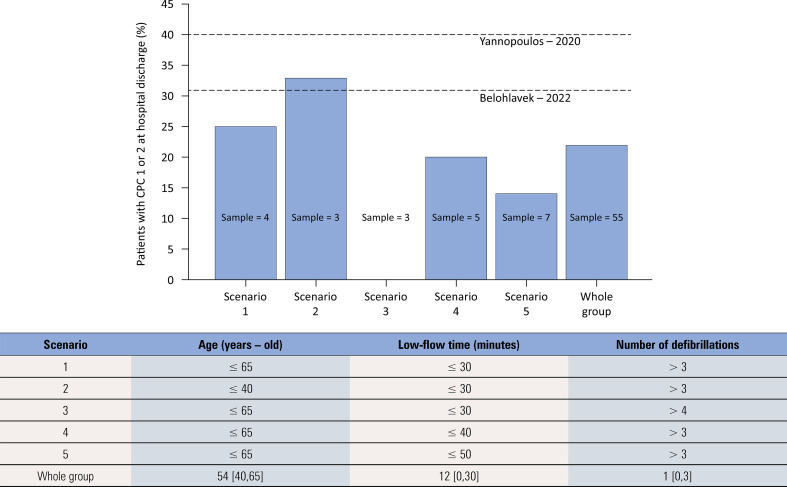
Percentage of patients with a favorable neurological outcome at the time of discharge among the 55 patients with cardiac arrest admitted to our hospital. All patients analyzed in the figure had an initial shockable rhythm. The sample denotes the number of patients who met the criteria for extracorporeal cardiopulmonary resuscitation (Scenario). Yannopoulos (2020) and Belohlavek (2022) denote the percentage of patients with the last reported favorable neurological outcome in the extracorporeal cardiopulmonary resuscitation group of both randomized trials. The values for the whole group are shown as medians [25-75^th^ percentiles]. CPC - cerebral performance category.

Tables 3S - 6S (Supplementary Material) provide an overview of the pooled characteristics of the randomized (n = 3), propensity score-matched/coarsened exact-matched (n = 14), logistic regression-adjusted (n = 6), and nonmatched/nonadjusted studies (n = 8). Publication bias was assessed using funnel plots, as shown in figures 4S - 6S (Supplementary Material).

### Sensitivity analyses

We performed sensitivity analyses for both the last reported favorable neurological outcome and the last reported survival, incorporating data from randomized, propensity score-matched, coarsened exact-matched, and logistic regression-adjusted studies. The results of these analyses are presented in figures 7S and 8S (Supplementary Material).

### Cardiac arrest registry

Our local cardiac arrest registry included 55 patients admitted to the emergency department (Tables 1 and 2). The median age of these patients was 54 years (interquartile range 40 - 65), and the overall survival rate was 18.2% (10/55 patients). All survivors had an initial shockable rhythm. To explore the potential impact of ECPR in our setting, we modeled several patient selection scenarios on the basis of age, low-flow time, initial rhythm, and the number of defibrillations (Figure 4). The criteria for patient enrollment based on these factors is shown in figures 9S and 10S (Supplementary Material).

**Table 1:** General characteristics of the 55 resuscitated patients according to survival

	Whole-group	Survivors	Non-survivors	p value*
Sample size	55	10	45	
Age (years)	54 [40 - 65]	57 [51 - 61]	53 [37 - 66]	0.896
Female	15 (27)	2 (20)	13 (29)	0.858
Estimated height (cm)	170 [160 -175]	173 [170 - 179]	170 [160 - 175]	0.223
Estimated weight (kg)	70 [65 - 80]	71 [66 - 80]	70 [60 - 80]	0.397
Comorbidities				
Arterial hypertension	18 (33)	4 (40)	14 (31)	0.866
Diabetes mellitus type 2	7 (13)	1 (10)	6 (13)	> 0.99
Dialytic chronic renal failure	1 (2)	0 (0)	1 (2)	> 0.99
Chronic renal failure	1 (2)	0 (0)	1 (2)	> 0.99
COPD	1 (2)	0 (0)	1 (2)	> 0.99
Coronary disease	6 (11)	3 (30)	3 (7)	0.114
CABG	2 (4)	2 (20)	0 (0)	0.034
Heart failure	1 (2)	0 (0)	1 (2)	> 0.99
Others	21 (38)	3 (30)	18 (40)	0.819
Neoplasm	2 (4)	0 (0)	2 (4)	> 0.99
Sepsis	10 (18)	1 (10)	9 (20)	0.773
Aortic valve disease	0 (0)	0 (0)	0 (0)	> 0.99
Neurological diseases	9 (16)	1 (10)	8 (20)	0.897
Acute neurological disorders	47 (86)	9 (90)	38 (84)	0.108
Cerebral hemorrhage	5 (9)	0 (0)	5 (11)	
Stroke	2 (4)	0 (0)	2 (4)	
Meningitis	1 (2)	1 (10)	0 (0)	
Organ dysfunctions	12 (22)	1 (10)	11 (24)	0.564
Hemodynamic	4 (7)	0 (0)	4 (9)	0.760
Respiratory	8 (15)	1 (10)	7 (16)	> 0.99
Hematological	2 (4)	0 (0)	2 (4)	> 0.99
Liver	1 (2)	0 (0)	1 (2)	> 0.99
Neurological	7 (13)	0 (0)	7 (16)	0.418
Renal	5 (9)	1 (10)	4 (9)	> 0.99
Deficits in instrumental/daily activities†	5 (9)	1 (10)	4 (9)	> 0.99
Food preparation	3 (6)	1 (10)	2 (4)	> 0.99
Bathing prepare	5 (9)	1 (10)	4 (9)	> 0.99
Choosing clothes	2 (4)	1 (10)	1 (2)	0.799
Cleaning	3 (6)	1 (10)	2 (4)	> 0.99
Continence	1 (2)	0 (0)	1 (2)	> 0.99
Changing clothes	4 (7)	1 (10)	3 (7)	> 0.99
Bathing	4 (7)	1 (10)	3 (7)	> 0.99
Financial care	4 (7)	1 (10)	3 (7)	> 0.99
Taking buses	4 (7)	1 (10)	3 (7)	> 0.99
Shopping	4 (7)	1 (10)	3 (7)	> 0.99
Eating	4 (7)	1 (10)	3 (7)	> 0.99
Phone calling	3 (6)	1 (10)	2 (4)	> 0.99
Taking medicines	3 (6)	1 (10)	2 (4)	> 0.99
Domestic activities	4 (7)	1 (10)	3 (7)	> 0.99

COPD - obstructive pulmonary disease; CABG - coronary artery bypass graft.

* These are p values from the comparison between survivors and non-survivors; † baseline deficits. Qualitative data are shown as occurrences and percentages. Quantitative data are shown as medians [25-75th percentiles].

**Table 2:** Characteristics of cardiac arrest and resuscitation according to survival

	Whole-group	Survivors	Non-survivors	p value*
Sample size	55	10	45	
OHCA	33 (60)	7 (70)	26 (58)	0.721
Witnessed cardiac arrest	46 (84)	9 (90)	37 (82)	0.897
Immediate CPR starting	41 (75)	8 (80)	33 (73)	0.971
Time-to-start CPR (minutes)†	6.5 [5.0 - 12.5]	14.0 [11.0 - 17.0]	5.0 [5.0 - 10.0]	0.380
Time-to-hospital admission (minutes)	28.0 [18.8 - 35.0]	30.0 [22.5 - 45.0]	26.0 [15.0 - 35.0]	0.302
Time-to-final outcome (minutes)	3.0 [0.4 - 35.5]	15.5 [8.8 - 23.3]	0.8 [0.4 - 48.5]	0.462
Initial cardiac rhythm				
Ventricular fibrillation	20 (36)	5 (50)	15 (33)	0.530
Ventricular tachycardia	5 (9)	0 (0)	5 (11)	0.619
Asystole	17 (31)	2 (20)	15 (33)	0.655
Pulseless electrical activity	13 (24)	3 (30)	10 (22)	0.911
Defibrillation proceeded	30 (55)	6 (60)	24 (53)	0.975
Number of countershocks/patient	3 [1,4]	3 [1,3]	3 [2,4]	0.491
Cardiac arrest possible etiology				
Acute coronary syndrome	18 (33)	5 (50)	13 (29)	0.361
Massive pulmonary embolism	2 (4)	0 (0)	2 (4)	> 0.99
Pneumothorax	1 (2)	0 (0)	1 (2)	> 0.99
Hypoxemia	7 (13)	2 (20)	5 (11)	0.812
Acidosis	5 (9)	1 (10)	4 (9)	> 0.99
Others	20 (36)	2 (20)	18 (40)	0.409
Brain death	1 (2)	0 (0)	1 (2)	> 0.99
Organ donation	1 (2)	0 (0)	1 (2)	> 0.99
CPC 1 or 2 at hospital discharge ‡	7 (13)	7 (70)	------	------

OHCA - out-of-hospital cardiac arrest; CPC - cerebral performance category; CPR - cardiopulmonary resuscitation. * These are p values from the comparison between survivors and non-survivors. † Data from 14 patients in which cardiopulmonary resuscitation was not immediately started. ‡ One patient was discharged with cerebral performance category 3. Qualitative data are shown as occurrences and percentages. Quantitative data are shown as medians [25-75th percentiles].

In the most inclusive scenario (age ≤ 65 years, low-flow time ≤ 60 minutes), 13 patients would have met the eligibility criteria for ECPR, with an estimated 30.8% expected to achieve a favorable neurological outcome at discharge. Under stricter criteria (age ≤ 65 years, low-flow time ≤ 45 minutes, and number of defibrillations ≥ 3), only 5 patients would have been eligible. Figure 4 illustrates the percentage of patients discharged with favorable neurological outcome, including the last reported outcomes for patients in the ECPR groups from the Yannopoulos^(1)^ and Belohlavek^(2)^ studies. These data suggest the potential benefit of ECPR if applied to similar patients in our cohort.

## DISCUSSION

In this meta-analysis of 31 studies, including 3 randomized clinical trials and 28 observational studies with structured matching, ECPR was associated with improved favorable neurological outcomes and improved survival in patients with refractory cardiac arrest. However, randomized trials, with a marginal result, did not demonstrate statistically significant benefits. However, the inclusion of propensity score-matched and coarsened exact-matched studies strengthened associations for both favorable neurological outcomes and survival. Our local cardiac arrest registry identified between 4 and 7 potential candidates for ECPR every six months, depending on the criteria used. The viability of the ECPR criteria analyzed in our cardiac arrest registry was based on the number of patients with favorable neurological outcomes at hospital discharge, which must be less than the number of patients with favorable neurological outcomes in the ECPR group of the main randomized trials; these findings reinforce the possibility of improved outcomes (Figure 4). The more conservative criteria (initial shockable rhythm, age ≤ 65 y, low-flow time ≤ 30 minutes, and number of defibrillations > 3) were met by four patients during the six months studied, and this group of patients had lower rates of favorable neurological outcome and survival at discharge than did patients in the ECPR group of the randomized trials. We plan to use these more conservative criteria in the first year of the program and reevaluate them at the end of this time.

Our systematic review, meta-analysis and TSA reinforced the current evidence supporting the use of ECPR to treat patients with OHCA and IHCA, showing a consistent reduction in intrahospital mortality and a high number of patients with favorable neurological outcomes.^(24)^ Second, the cardiac arrest registry did not capture the majority of patients with cardiac conditions. However, the number of patients who met the ECPR criteria justifies beginning our program. Additionally, although we aimed to include all patients with cardiac arrest, there was no formal compliance check to ensure complete data collection. Nonetheless, the primary objective was to estimate a minimum frequency of eligible patients, which was achieved. Finally, our sample of patients with cardiac arrest likely reflects a population with better outcomes, as it predominantly includes patients with OHCA who were transported to the hospital. This suggests that the decision to transport these patients was likely based on more favorable clinical scenarios, which may explain the relatively high baseline survival rate even without the use of ECPR.

Among the three randomized ECPR trials, all included patients with OHCA from high-income countries; furthermore, the INCEPTION trial reported a neutral result.^(25)^ The INCEPTION trial has several points to be considered: only 66% of patients allocated to the ECPR group received the intervention; the survival of the control group was double that expected, despite the very fast arrival of the emergency medical system team; the low-flow time was significantly higher than that of the other two randomized trials (74 minutes *versus* 59 min in the ARREST trial^(1)^ and 58 minutes in the PRAGUE trial^(2)^) and the emergency room teams were not blinded at the time of patient admission and could withdraw patients. The INCEPTION trial authors considered the trial to be underpowered, mainly due to the high survival of the control group and the positive finding of longer time persistence of ventricular malignant arrhythmias in the ECPR group.^(26)^ Taking this information into consideration, the same Netherlands group that ran the INCEPTION trial is expanding the use of ECPR to nationwide coverage through the helicopter emergency medical system in a nonrandomized stepped-wedge design study, the ON-SCENE trial.^(27)^ Furthermore, studies indicate that favorable ECPR outcomes are associated with centers using ECPR to treat more than 12 patients annually.^(28)^ The high-expertise centers in the ARREST and PRAGUE OHCA trials achieved rapid door-to-ECMO times, whereas the INCEPTION trial involved less-experienced centers, resulting in longer times and neutral findings.^(1,2,23)^These observations underscore the importance of developing dedicated ECMO centers to improve clinical outcomes.

The criteria for ECPR inclusion vary widely among ECMO centers and depend on several factors, such as geographic location (rural vs. metropolitan), hospital specialty, and emergency medical system protocols.^(29)^ Understanding the specific patient population and survival rates under standard care is essential before implementing interventional strategies such as ECPR.

Extracorporeal membrane oxygenation is cost effective in high-income countries.^(30)^ However, cost effectiveness is a potential problem in implementing ECMO centers in middle-income countries. In Brazil, a single-center cost-effectiveness analysis revealed a safe profile once continued education is concomitantly applied.^(31)^ Despite this fact and the consistent current evidence on ECMO respiratory support, the Brazilian health technology assessment (HTA) committee has not incorporated ECMO technology into the Brazilian public health system due to economic concerns, mainly related to inequity resulting in increased risk in Brazilian regions.^(7)^Equity has recently become a major public health problem for Brazilian neonates, as a structural imbalance has generated less support for poorer regions; this is known as inverse equity.^(32)^ During the COVID-19 pandemic, the misapplication of the equity concept by the Brazilian HTA committee also resulted in inverse equity between the public health system (*Sistema Único de Saúde* [SUS]) and the private health system, as ECMO support was provided to more patients in the less-crowded private health system than in the crowded SUS (these data include all humans in Brazilian territory, including foreigners).^(33)^ Equity is a core principle of the SUS; therefore, policy advocacy to increase funding for ECMO programs can emphasize the long-term positive effects of these programs for patient outcomes, regardless of patient social class and economic status. Additionally, exploring public-private partnerships may help fund ECMO initiatives, alleviating financial burdens on public health care systems.^(34)^

Our cardiac arrest registry revealed a high number of patients with acute neurologic disorders. This occurred because the cardiological emergency room of the hospital is close to the general emergency room but physically in another location. Therefore, this registry is important for characterizing the plausibility of an ECPR program in the general emergency room.

## CONCLUSION

In this study, the use of extracorporeal cardiopulmonary resuscitation to treat patients with refractory cardiac arrest was associated with an increased number of patients with favorable neurological outcomes and improved survival. Our local cardiac arrest registry data indicate the plausibility of an extracorporeal cardiopulmonary resuscitation program in our hospital. Using the more conservative criteria (initial shockable rhythm, age ≤ 65 years, low-flow time ≤ 30 minutes, and number of defibrillations > 3), we expect to include 4 patients within the first six months of beginning an extracorporeal cardiopulmonary resuscitation program.
