# Supplementary material for: Feasibility of implementing extracorporeal cardiopulmonary resuscitation in a middle-income country: systematic review and cardiac arrest case series
Source: Crit Care Sci. 2025 May 6;37:e20250320. doi: 10.62675/2965-2774.20250320 (PMC12094697; doi:10.62675/2965-2774.20250320)
Supplement: Supplementary file 1 [file 2965-2774-ccsci-37-e20250320-suppl01.pdf]

Critical Care Science
https://doi.org/10.62675/2965-2774.20250320

SUPPLEMENTARY MATERIAL

Feasibility of implementing extracorporeal cardiopulmonary resuscitation in a middle-income country: systematic review and cardiac arrest case series

Gabriel Afonso Dutra Kreling[1], Pedro Vitale Mendes[1], Luis Carlos Maia Cardozo Junior[1], Karina Turaça Kasahaya[2], Marcelo Park[1], Ludhmila Abrahão Hajjar[1], Ian Ward A. Maia[2]

METHODS

The search strategy used in the systematic review was:

Population:

cardiopulmonary resuscitation ***or*** CPR ***or*** cardiac life support ***or*** heart arrest ***or*** cardiac arrest ***or*** cardiopulmonary arrest

and

Intervention:

extracorporeal membrane oxygenation ***or*** extracorporeal life support ***or*** ECMO ***or*** ECLS ***or*** extracorporeal cardiopulmonary resuscitation ***or*** extracorporeal circulation ***or*** ECPR

The full search strategies used, divided by database, was:

Web of Science

No	Search	Results
1.	"Population":<br>TS=(cardiopulmonary resuscitation OR CPR OR Cardiac life support OR Heart arrest OR Cardiopulmonary arrest OR ECPR OR Extracorporeal cardiopulmonary resuscitation)	64,518
2.	"Intervention":<br>TS=(Extracorporeal Membrane Oxygenation OR Extracorporeal Life Support OR ECMO OR ECLS OR Extracorporeal cardiopulmonary resuscitation OR Extracorporeal Circulat*)	34,373
	#1 AND #2	5,279
	Studies after 2000	5,004

Science Direct

No	Search	Results
1.	"Population":<br>Title, abstract, Keywords: (cardiopulmonary resuscitation OR CPR OR Cardiac life support OR Heart arrest OR Cardiopulmonary arrest OR ECPR OR Extracorporeal cardiopulmonary resuscitation)	21,325
2.	"Intervention":<br>Title, abstract, keywords: (Extracorporeal Membrane Oxygenation OR Extracorporeal Life Support OR ECMO OR ECLS OR Extracorporeal cardiopulmonary resuscitation OR Extracorporeal Circulation)	14,470
	#1 AND #2	39

This is an open access article under the CC BY license (https://creativecommons.org/licenses/by/4.0/).

Pubmed

No	Search	Results
1.	"Population":<br>Cardiopulmonary resuscitation[MeSH] OR CPR[Title/Abstract] OR<br>Cardiopulmonary resuscitation[Title/Abstract] OR Cardiac life support[Title/Abstract] OR Heart Arrest[MeSH] OR Cardiac arrest[Title/Abstract] OR Cardiopulmonary arrest [Title/Abstract] OR ECPR[Title/Abstract] OR Extracorporeal cardiopulmonary resuscitation[Title/Abstract]	93,225
2.	"Intervention":<br>Extracorporeal Membrane Oxygenation[MeSH] OR Extracorporeal Membrane Oxygenation[Title/Abstract] OR Extracorporeal Membrane Oxygenat*[Title/Abstract] OR Extracorporeal Life Support[Title/Abstract] OR ECMO[Title/Abstract] OR<br>ECLS[Title/Abstract] OR Extracorporeal Circulat*[Title/Abstract] OR<br>"Extracorporeal cardiopulmonary resuscitation"[Title/Abstract]	32,249
	#1 AND #2	3,556
	Studies after 2000	3,327

LILACS

No	Search	Results
1.	"Population":<br>(cardiopulmonary resuscitation OR CPR OR Cardiac life support OR Heart arrest OR Cardiopulmonary arrest OR ECPR OR Extracorporeal cardiopulmonary resuscitation)	27,230
2.	"Intervention":<br>(Extracorporeal Membrane Oxygenation OR Extracorporeal Life Support OR ECMO OR ECLS OR Extracorporeal cardiopulmonary resuscitation OR Extracorporeal Circulat$)	1,756
	#1 AND #2	935

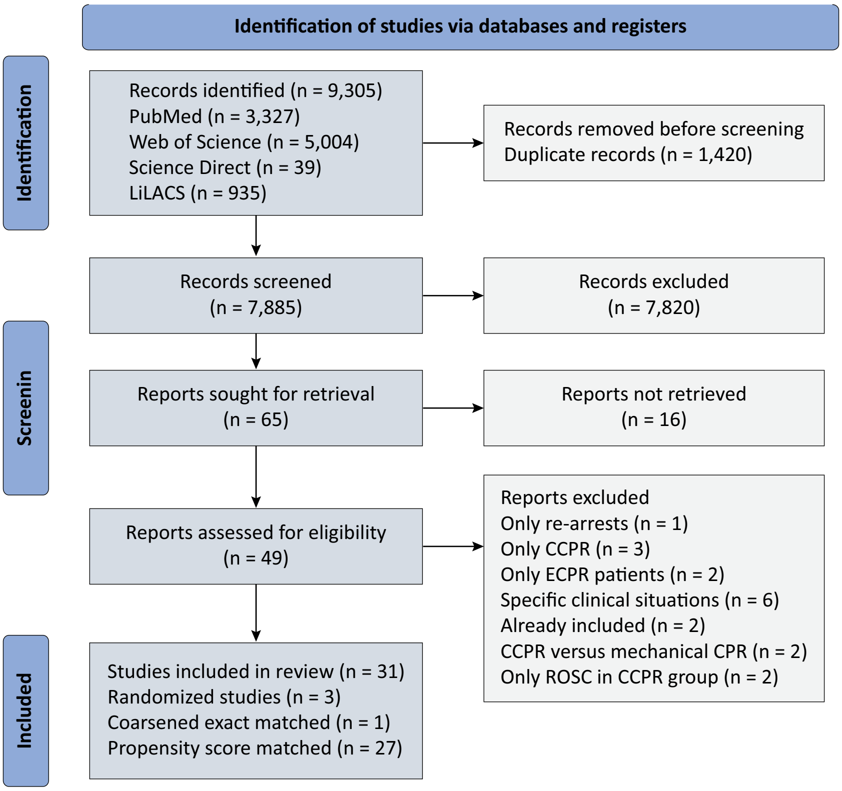

Figure 1S - Preferred reporting items for systematic reviews and meta-analyses (PRISMA) flowchart.
CCPR - conventional cardiopulmonary resuscitation; ECPR - extracorporeal cardiopulmonary resuscitation; CPR - cardiopulmonary resuscitation; ROSC - return of spontaneous circulation.

RESULTS

Table 1S - Characteristics of all studies analyzed in the systematic review

Author	Country	Adjustment/ matching	ECPR criteria	Cardiac arrest place	Sample size	Males	Age	Initial shockable rhythm	Low-flow time	CPC 1 or 2	ECPR survival	CCPR survival
Chen et al.[1]	Taiwan	Propensity score	> 18 yo Refractory cardiac arrest	IHCA	ECPR 46 CCPR 46	ECPR 39 CCPR 40	ECPR 57 ± 14 CCPR 55 ± 14	ECPR 21 CCPR 19	Not reported	Hospital ECPR 14 CCPR 7	Hospital = 15 30 days = NR 3 months = NR 6 months = NR 1 year = NR Last reported = 15	Hospital = 8 30 days = 3 months = 6 months = 1 year = Last reported = 8
Shin et al.[2]	Korea	Propensity score	> 18 yo Refractory cardiac arrest	IHCA	ECPR 60 CCPR 60	ECPR 36 CCPR 41	ECPR 60.8 ± 14,5 CCPR 60.5 ± 14.5	ECPR 13 CCPR 13	ECPR 38.8 ± 10 CCPR 38.1 ± 15	6 months ECPR 14 CCPR 3	Hospital = NR 30 days = NR 3 months = NR 6 months = NR 1 year = 13 Last reported = 13	Hospital = 30 days = 3 months = 6 months = 1 year = 5 Last reported = 5
Maekawa et al.[3]	Japan	Propensity score	> 18 yo Refractory cardiac arrest	OHCA	ECPR 24 CCPR 24	ECPR 19 CCPR 19	ECPR 56 ± 11.82 CCPR 58.33 ± 14.19	ECPR 13 CCPR 14	ECPR 52.67 ± 18.13 CCPR 53.33 ± 17.34	3 months ECPR 7 CCPR 2	Hospital = NR 30 days = 9 3 months = NR 6 months = NR 1 year = NR Last reported = 9	Hospital = 30 days = 2 3 months = 6 months = 1 year = Last reported = 2
Chou et al.[4]	Taiwan	No	> 18 yo Refractory cardiac arrest	IHCA	ECPR 43 CCPR 23	ECPR 40 CCPR 17	ECPR 60 ± 40 CCPR 69 ± 69	ECPR 26 CCPR 9	NR	NR	Hospital = NR 30 days = NR 3 months = NR 6 months = NR 1 year = 15 Last reported = 15	Hospital = 30 days = 3 months = 6 months = 1 year = 5 Last reported = 5
Sakamoto et al.[5]	Japan	No	> 18 yo Refractory cardiac arrest	OHCA	ECPR 260 CCPR 194	ECPR 235 CCPR 172	ECPR 56.3 ± 15 CCPR 58.1 ± 58.1	Not clear	NR	6 months ECPR 29 CCPR 5	Hospital = NR 30 days = NR 3 months = NR 6 months = 57 1 year = NR Last reported = 57	Hospital = 30 days = 3 months = 6 months = 6 1 year = Last reported = 6
Lee et al.[6]	Korea	Regression adjustment	> 18 yo Refractory cardiac arrest	OHCA	ECPR 81 CCPR 874	ECPR 56 CCPR 564	ECPR 59 ± 18.6 CCPR 63.5 ± 17	ECPR 34 CCPR 129	ECPR 41.3 ± 29.4 CCPR 31 ± 24.5	NR	Hospital = NR 30 days = NR 3 months = NR 6 months = NR 1 year = 16 Last reported = 16	Hospital = NR 30 days = NR 3 months = NR 6 months = NR 1 year = 192 Last reported = 192
Siao et al.[7]	Taiwan	Regression adjustment	> 18 yo Refractory cardiac arrest	OHCA and IHCA	ECPR 20 CCPR 40	ECPR 18 CCPR 28	ECPR 54.55 ± 11.94 CCPR 60.28 ± 15	Not clear	ECPR 34.38 ± 18 CCPR 69.9 ± 18	1 year ECPR 8 CCPR 3	Hospital = 10 30 days = NR 3 months = NR 6 months = NR 1 year = 10 Last reported = 10	Hospital = 11 30 days = NR 3 months = NR 6 months = NR 1 year = 10 Last reported = 10
Blumenstein et al.[8]	Germany	Propensity score	> 18 yo Refractory cardiac arrest	OHCA	ECPR 52 CCPR 52	ECPR 31 CCPR 31	ECPR 68.3 ± 17.46 CCPR 73 ± 7.62	ECPR 16 CCPR 15	ECPR 37,3 ± 11.4 CCPR 21.83 ± 26.3	1 year ECPR 10 CCPR 6	Hospital = NR 30 days = NR 3 months = NR 6 months = NR 1 year = 11 Last reported = 11	Hospital = NR 30 days = NR 3 months = NR 6 months = 7 1 year = NR Last reported = 7

Continue...

...continuation

Choi et al.[9]	Korea	No	> 18 yo<br>Refractory<br>cardiac<br>arrest	OHCA	ECPR<br>10<br>CCPR<br>50	ECPR<br>7<br>CCPR<br>38	ECPR<br>58.9 ± 11.2<br>CCPR<br>59.2 ± 59.2	ECPR<br>3<br>CCPR<br>13	ECPR<br>18 ± 18<br>CCPR<br>18.8 ± 18.8	30 days<br><br>ECPR<br>3<br>CCPR<br>2	Hospital = 3<br>30 days = 3<br>3 months = NR<br>6 months = NR<br>1 year = NR<br>Last reported = 3	Hospital = NR<br>30 days = 4<br>3 months = NR<br>6 months = NR<br>1 year = NR<br>Last reported<br>= 4
Choi et al.[10]	Korea	Propensity<br>score	> 18 yo<br>Refractory<br>cardiac<br>arrest st	OHCA	ECPR<br>320<br>CCPR<br>320	ECPR<br>258<br>CCPR<br>142	ECPR<br>56.33 ± 17.13<br>CCPR<br>57.67 ± 15.64	ECPR<br>93<br>CCPR<br>88	ECPR<br>19.33 ± 9.68<br>CCPR<br>19.33 ± 9.68	Hospital<br><br>ECPR<br>29<br>CCPR<br>19	Hospital = 57<br>30 days = NR<br>3 months = NR<br>6 months = NR<br>1 year = NR<br>Last reported = 57	Hospital = 52<br>30 days = NR<br>3 months = NR<br>6 months = NR<br>1 year = NR<br>Last reported<br>= 52
Nakashima et al.[11]	Japan	Regression<br>adjustment	> 18 yo<br>Refractory<br>cardiac<br>arrest	OHCA	ECPR<br>250<br>CCPR<br>157	ECPR<br>227<br>CCPR<br>139	ECPR<br>58 ± 14.17<br>CCPR<br>59.67 ± 12.72	ECPR<br>127<br>CCPR<br>60	NR	6<br>months<br><br>ECPR<br>25<br>CCPR<br>2	Hospital = NR<br>30 days = NR<br>3 months = NR<br>6 months = 41<br>1 year = NR<br>Last reported = 41	Hospital = NR<br>30 days = NR<br>3 months = NR<br>6 months = 4<br>1 year = NR<br>Last reported<br>= 4
Shinar et al.[12]	United<br>States	Regression<br>adjustment	> 18 yo<br>Refractory<br>cardiac<br>arrest	OHCA	ECPR<br>25<br>CCPR<br>183	ECPR<br>22<br>CCPR<br>109	ECPR<br>57.9 ± 13.7<br>CCPR<br>68.7 ± 12	ECPR<br>14<br>CCPR<br>51	ECPR<br>59 ± 11.79<br>CCPR<br>34.3 ± 24.6	NR	Hospital = 6<br>30 days = NR<br>3 months = NR<br>6 months = NR<br>1 year = NR<br>Last reported = 6	Hospital = 26<br>30 days = NR<br>3 months = NR<br>6 months = NR<br>1 year = NR<br>Last reported<br>= 26
Patel et al.[13]	United<br>States	Coarsened<br>exact<br>matching	> 18 yo<br>Refractory<br>cardiac<br>arrest	OHCA	ECPR<br>534<br>CCPR<br>534	ECPR<br>196<br>CCPR<br>196	Not clear	ECPR<br>224<br>CCPR<br>236	NR	NR	Hospital = 321<br>30 days = NR<br>3 months = NR<br>6 months = NR<br>1 year = NR<br>Last reported =<br>321	Hospital = 305<br>30 days = NR<br>3 months = NR<br>6 months = NR<br>1 year = NR<br>Last reported<br>= 305
Patricio et al.[14]	Belgium	Propensity<br>score	> 18 yo<br>Refractory<br>cardiac<br>arrest	OHCA e<br>IHCA	ECPR<br>80<br>CCPR<br>80	ECPR<br>59<br>CCPR<br>49	ECPR<br>57 ± 14<br>CCPR<br>57 ± 57	ECPR<br>24<br>CCPR<br>23	ECPR<br>54 ± 54<br>CCPR<br>54 ± 54	3<br>months<br><br>ECPR<br>17<br>CCPR<br>9	Hospital = 18<br>30 days = NR<br>3 months = NR<br>6 months = NR<br>1 year = NR<br>Last reported = 18	Hospital = 14<br>30 days = NR<br>3 months = NR<br>6 months = NR<br>1 year = NR<br>Last reported<br>= 14
Bartos et al.[15]	United<br>States	Regression<br>adjustment	> 18 yo<br>Refractory<br>cardiac<br>arrest	OHCA	ECPR<br>160<br>CCPR<br>974	ECPR<br>126<br>CCPR<br>528	ECPR<br>57 ± 13<br>CCPR<br>59 ± 59	ECPR<br>100<br>CCPR<br>654	ECPR<br>60 ± 60<br>CCPR<br>35 ± 35	Hospital<br><br>ECPR<br>52<br>CCPR<br>148	Hospital = NR<br>30 days = NR<br>3 months = NR<br>6 months = NR<br>1 year = NR<br>Last reported<br>= NR	Hospital = NR<br>30 days = NR<br>3 months = NR<br>6 months = NR<br>1 year = NR<br>Last reported<br>= NR
Bougouin et al.[16]	France	Regression<br>adjustment	> 18 yo<br>Refractory<br>cardiac<br>arrest	OHCA	ECPR<br>525<br>CCPR<br>12,666	ECPR<br>442<br>CCPR<br>8,480	ECPR<br>50 ± 13<br>CCPR<br>66 ± 66	ECPR<br>358<br>CCPR<br>12,666	NR	NR	Hospital = 44<br>30 days = NR<br>3 months = NR<br>6 months = NR<br>1 year = NR<br>Last reported = 44	Hospital = 109<br>30 days = NR<br>3 months = NR<br>6 months = NR<br>1 year = NR<br>Last reported<br>= 109

Continue...

...continuation

Kim et al.[17]	Korea	Propensity score	> 18 yo Refractory cardiac arrest	OHCA	ECPR 3,826 CCPR 3,826	ECPR 2,603 CCPR 2,826	ECPR 59.8 ± 14.9 CCPR 59.7 ± 59.7	ECPR 2,413 CCPR 1,279	NR	NR	Hospital = NR<br>30 days = NR<br>3 months = NR<br>6 months = NR<br>1 year = 682<br>Last reported = 682	Hospital = NR<br>30 days = NR<br>3 months = NR<br>6 months = NR<br>1 year = 431<br>Last reported = 431
Nakajima et al.[18]	Japan	No	> 18 yo Refractory cardiac arrest	OHCA	ECPR 5,612 CCPR 212,295	ECPR 4,425 CCPR 120,923	ECPR 61 ± 15.57 CCPR 76.6 ± 13.3	ECPR 3,004 CCPR 8,068	NR	Hospital<br>ECPR 696 CCPR 3,397	Hospital = 898<br>30 days = NR<br>3 months = NR<br>6 months = NR<br>1 year = NR<br>Last reported = 898	Hospital = 5732<br>30 days = NR<br>3 months = NR<br>6 months = NR<br>1 year = NR<br>Last reported = 5,732
Shin et al.[19]	Korea	No	> 18 yo Refractory cardiac arrest	OHCA	ECPR 30 CCPR 40	ECPR 25 CCPR 30	ECPR 60 ± 12.45 CCPR 60.67 ± 14.61	ECPR 18 CCPR 19	ECPR 49.3 ± 15 CCPR 51.3 ± 15	6 months<br>ECPR 10 CCPR 2	Hospital = NR<br>30 days = NR<br>3 months = NR<br>6 months = 10<br>1 year = NR<br>Last reported = 10	Hospital = NR<br>30 days = NR<br>3 months = NR<br>6 months = 2<br>1 year = NR<br>Last reported = 2
Yannopoulos et al.[20]	United States	Randomized	18 – 75 yo Shockable rhythm with > 3 shocks Time to hosp < 30 min	OHCA	ECPR 15 CCPR 15	ECPR 14 CCPR 11	ECPR 59 ± 10 CCPR 58 ± 58	ECPR 15 CCPR 15	ECPR 30 ± 30 CCPR 30 ± 30	6 months<br>ECPR 6 CCPR 1	Hospital = 6<br>30 days = NR<br>3 months = NR<br>6 months = 6<br>1 year = NR<br>Last reported = 6	Hospital = NR<br>30 days = NR<br>3 months = NR<br>6 months = 0<br>1 year = NR<br>Last reported = 0
Yoshida et al.[21]	Japan	No	> 18 yo Refractory cardiac arrest	OHCA	ECPR 38 CCPR 493	ECPR 27 CCPR 334	ECPR 60.6 ± 15.6 CCPR 70.7 ± 18	Not clear	NR	3 months<br>ECPR 5 CCPR 8	Hospital = NR<br>30 days = NR<br>3 months = 6<br>6 months = NR<br>1 year = NR<br>Last reported = 6	Hospital = NR<br>30 days = NR<br>3 months = 18<br>6 months = NR<br>1 year = NR<br>Last reported = 18
Alm-Kruse et al.[22]	Norway	No	> 18 yo Refractory cardiac arrest	OHCA	ECPR 100 CCPR 48	ECPR 84 CCPR 41	ECPR 55.67 ± 9.03 CCPR 57.67 ± 8.41	ECPR 100 CCPR 48	NR	Hospital<br>ECPR 30 CCPR 21	Hospital = NR<br>30 days = 37<br>3 months = NR<br>6 months = NR<br>1 year = NR<br>Last reported = 37	Hospital = NR<br>30 days = 21<br>3 months = NR<br>6 months = NR<br>1 year = NR<br>Last reported = 21
Belohlavek et al.[23]	Czech Republic	Randomized	18 – 65 yo Witnessed CA > 5 min of CPR	OHCA	ECPR 124 CCPR 132	ECPR 102 CCPR 110	ECPR 57.67 ± 13.5 CCPR 56.33 ± 13.49	ECPR 72 CCPR 84	ECPR 54 ± 12 CCPR 62 ± 16.49	Hospital<br>ECPR 38 CCPR 24	Hospital = NR<br>30 days = 54<br>3 months = NR<br>6 months = NR<br>1 year = NR<br>Last reported = 54	Hospital = NR<br>30 days = 45<br>3 months = NR<br>6 months = NR<br>1 year = NR<br>Last reported = 45
Ben-Hamouda et al.[24]	Swiss	No	> 18 yo Refractory cardiac arrest	Not clear	ECPR 50 CCPR 397	ECPR 37 CCPR 275	ECPR 53.07 ± 21.6 CCPR 65.33 ± 14.88	ECPR 26 CCPR 189	NR	3 months<br>ECPR 13 CCPR 163	Hospital = NR<br>30 days = NR<br>3 months = 20<br>6 months = NR<br>1 year = NR<br>Last reported = 20	Hospital = NR<br>30 days = NR<br>3 months = 211<br>6 months = NR<br>1 year = NR<br>Last reported = 211

Continue...

...continuation

Fukushima et al.[25]	Japan	Propensity score	> 18 yo<br>Refractory cardiac arrest	OHCA	ECPR<br>149<br>CCPR<br>149	ECPR<br>114<br>CCPR<br>122	ECPR<br>58 ± 12<br>CCPR<br>56.9 ± 56.9	ECPR<br>149<br>CCPR<br>149	ECPR<br>49.3 ± 17<br>CCPR<br>37.5 ± 37.5	Hospital<br><br>ECPR<br>7<br>CCPR<br>3	Hospital = NR<br>30 days = 22<br>3 months = NR<br>6 months = NR<br>1 year = NR<br>Last reported = 22	Hospital = NR<br>30 days = 10<br>3 months = NR<br>6 months = NR<br>1 year = NR<br>Last reported = 10
Jeong et al.[26]	Korea	Propensity score	> 18 yo<br>Refractory cardiac arrest	OHCA	ECPR<br>271<br>CCPR<br>271	ECPR<br>211<br>CCPR<br>206	ECPR<br>58 ± 13.42<br>CCPR<br>58.33 ± 17.89	ECPR<br>162<br>CCPR<br>157	NR	Hospital<br><br>ECPR<br>10<br>CCPR<br>44	Hospital = NR<br>30 days = 45<br>3 months = NR<br>6 months = NR<br>1 year = NR<br>Last reported = 45	Hospital = NR<br>30 days = 53<br>3 months = NR<br>6 months = NR<br>1 year = NR<br>Last reported = 53
Choi et al.[27]	Korea	Propensity score	> 18 yo<br>Refractory cardiac arrest	OHCA	ECPR<br>458<br>CCPR<br>1,832	ECPR<br>374<br>CCPR<br>1,474	ECPR<br>55.5 ± 14.1<br>CCPR<br>56.4 ± 56.4	ECPR<br>271<br>CCPR<br>1,052	NR	Hospital<br><br>ECPR<br>47<br>CCPR<br>127	Hospital = 67<br>30 days = NR<br>3 months = NR<br>6 months = NR<br>1 year = NR<br>Last reported = 67	Hospital = 254<br>30 days = NR<br>3 months = NR<br>6 months = NR<br>1 year = NR<br>Last reported = 254
Ko et al.[28]	Korea	Propensity score	> 18 yo<br>Refractory cardiac arrest	OHCA	ECPR<br>139<br>CCPR<br>471	ECPR<br>118<br>CCPR<br>399	ECPR<br>55.33 ± 14.98<br>CCPR<br>55 ± 16.36	ECPR<br>65<br>CCPR<br>111	NR	Hospital<br><br>ECPR<br>11<br>CCPR<br>37	Hospital = 16<br>30 days = NR<br>3 months = NR<br>6 months = NR<br>1 year = NR<br>Last reported = 16	Hospital = 70<br>30 days = NR<br>3 months = NR<br>6 months = NR<br>1 year = NR<br>Last reported = 70
Suverein et al.[29]	Netherlands	Randomized	18 – 70 yo<br>Witnessed CA<br>> 15 min of CPR	OHCA	ECPR<br>70<br>CCPR<br>70	ECPR<br>63<br>CCPR<br>57	ECPR<br>54 ± 12<br>CCPR<br>57 ± 57	ECPR<br>69<br>CCPR<br>63	ECPR<br>74.6 ± 18.1<br>CCPR<br>49 ± 20	6 months<br><br>ECPR<br>14<br>CCPR<br>10	Hospital = 14<br>30 days = NR<br>3 months = NR<br>6 months = NR<br>1 year = NR<br>Last reported = 14	Hospital = 13<br>30 days = NR<br>3 months = NR<br>6 months = NR<br>1 year = NR<br>Last reported = 13
Sun et al.[30]	China	Propensity score	> 18 yo<br>Refractory cardiac arrest	OHCA	ECPR<br>22<br>CCPR<br>22	Not clear	ECPR<br>60 ± 11.6<br>CCPR<br>61.9 ± 15	ECPR<br>13<br>CCPR<br>9	NR	Hospital<br><br>ECPR<br>4<br>CCPR<br>1	Hospital = 4<br>30 days = NR<br>3 months = 3<br>6 months = NR<br>1 year = NR<br>Last reported = 3	Hospital = 1<br>30 days = NR<br>3 months = 1<br>6 months = NR<br>1 year = NR<br>Last reported = 1
Okada et al.[31]	Japan	Propensity score	> 18 yo<br>Refractory cardiac arrest	OHCA	ECPR<br>1,283<br>CCPR<br>1,283	ECPR<br>1,068<br>CCPR<br>1,056	ECPR<br>58.33 ± 14.84<br>CCPR<br>59 ± 16.33	ECPR<br>913<br>CCPR<br>913	NR	30 days<br><br>ECPR<br>126<br>CCPR<br>104	Hospital = NR<br>30 days = 269<br>3 months = NR<br>6 months = NR<br>1 year = NR<br>Last reported = 269	Hospital = NR<br>30 days = 158<br>3 months = NR<br>6 months = NR<br>1 year = NR<br>Last reported = 158

ECPR - extracorporeal cardiopulmonary resuscitation; CPC - cerebral performance category; CCPR - conventional cardiopulmonary resuscitation; IHCA - in-hospital cardiac arrest; NR - not reported.; OHCA - out-of-hospital cardiac arrest.z

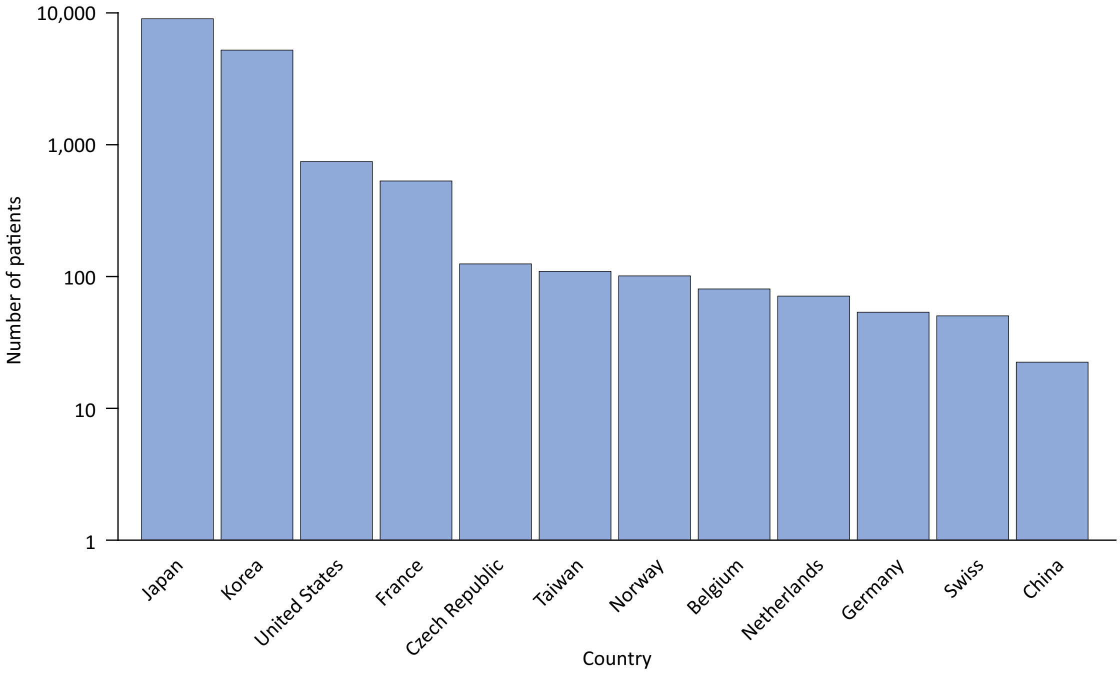

Figure 2S - Number of patients analyzed in the systematic review according to the country of origin.
Attention to the Log_{10} transformed the Y axis scale.

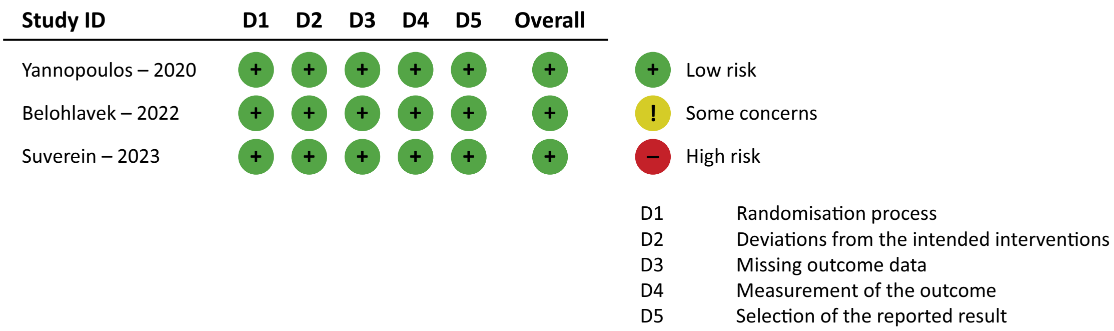

Figure 3S - ROB-2 risk of bias of the enrolled randomized studies in the systematic review.
D - day.

Table 2S - ROBINS-1 risk of bias of the enrolled in the systematic review observational paired studies

Study	Bias due to confounding	Bias in selection of participants into the study	Bias in classification of interventions	Bias due to deviations from intended interventions	Bias due to missing data	Bias in measurement of outcomes	Bias in selection of the reported result	Overall bias
Chen et al.[1]	Moderate	Moderate	Low	Low	Low	Moderate	Low	Serious
Shin et al.[2]	Moderate	Moderate	Low	Low	Low	Moderate	Low	Serious
Maekawa et al.[3]	Moderate	Moderate	Low	Low	Low	Moderate	Low	Serious
Chou et al.[4]	Moderate	Moderate	Low	Low	Low	Moderate	Low	Serious
Sakamoto et al.[5]	Moderate	Moderate	Low	Low	Low	Low	Low	Serious
Lee et al.[6]	Moderate	Moderate	Low	Low	Low	Low	Low	Serious
Siao et al.[7]	Moderate	Moderate	Low	Low	Low	Low	Low	Serious
Blumenstein et al.[8]	Moderate	Moderate	Low	Low	Low	Moderate	Low	Serious
Choi et al.[9]	Moderate	Moderate	Low	Low	Low	Moderate	Low	Serious
Choi et al.[10]	Moderate	Moderate	Moderate	Low	Low	Low	Low	Serious
Nakashima et al.[11]	Moderate	Moderate	Low	Low	Low	Low	Low	Serious
Shinar et al.[12]	Moderate	Moderate	Low	Low	Low	Low	Low	Serious
Patel et al.[13]	Moderate	Moderate	Low	Low	Low	Moderate	Low	Serious
Patricio et al.[14]	Moderate	Moderate	Low	Low	Low	Moderate	Low	Serious
Bartos et al.[15]	Moderate	Moderate	Low	Low	Low	Low	Low	Serious
Bougouin et al.[16]	Moderate	Moderate	Low	Low	Low	Low	Low	Serious
Kim et al.[17]	Moderate	Moderate	Low	Low	Low	Moderate	Low	Serious
Nakajima et al.[18]	Moderate	Moderate	Moderate	Low	Low	Low	Low	Serious
Shin et al.[19]	Moderate	Moderate	Moderate	Low	Low	Low	Low	Serious
Yoshida et al.[21]	Moderate	Moderate	Moderate	Low	Low	Low	Low	Serious
Alm-Kruse et al.[22]	Moderate	Moderate	Moderate	Low	Low	Low	Low	Serious
Ben-Hamouda et al.[24]	Moderate	Moderate	Moderate	Low	Low	Low	Low	Serious
Fukushima et al.[25]	Moderate	Moderate	Low	Low	Low	Moderate	Low	Serious
Jeong et al.[26]	Moderate	Moderate	Low	Low	Low	Moderate	Low	Serious
Choi et al.[27]	Moderate	Moderate	Low	Low	Low	Moderate	Low	Serious
Ko et al.[28]	Moderate	Moderate	Low	Low	Low	Moderate	Low	Serious
Sun et al.[30]	Moderate	Moderate	Low	Low	Low	Moderate	Low	Serious
Okada et al.[31]	Moderate	Moderate	Low	Low	Low	Moderate	Low	Serious

Table 3S - Pooled characteristics of the patients and cardiac arrests of the three randomized studies

Characteristic	ECPR			Conventional CPR	
	Number of studies	Sample size	Pooled mean/ incidence (95%CI)	Sample size	Pooled mean/ incidence (95%CI)
Age (years)	3	209	57 (54 – 59)	209	57 (54 – 59)
Male - %	3	209	86 (77 – 92)	209	82 (76 – 87)
Cardiac arrest data					
OHCA - %	3	209	100	209	100
Acute coronary syndrome - %	3	209	62 (40 – 80)	209	61 (34 – 84)
Pulmonary embolism - %	3	209	5 (0 – 25)	209	4 (0 – 32)
Shockable rhythm - %	3	209	92 (47 – 99)	209	85 (55 – 96)
Low-flow time (minutes)*	3	209	54 (29 – 78)	209	54 (29 – 78)
Target temperature control - %	3	209	99 (95 – 100)	209	97 (82 – 100)
Last CPC 1 or 2 reported	3	209	28 (20 – 38)	209	16 (12 – 22)
Last survival reported - %	3	209	43 (35 – 52)	209	22 (1 – 42)

ECPR - extracorporeal cardiopulmonary resuscitation; CPR - cardiopulmonary resuscitation; 95%CI - 95% confidence interval; OHCA - out-of-hospital cardiac arres; CPC - cerebral performance category. * The time until extracorporeal membrane oxygenation blood flow or return of spontaneous circulation.

Table 4S - Pooled characteristics of patients and cardiac arrests of the fourteen propensity score or coarsened exact matched studies

Characteristic	ECPR			Conventional CPR	
	Number of studies	Sample size	Pooled mean/ incidence (95%CI)	Sample size	Pooled mean/ incidence (95%CI)
Age (years)	13	6,675	58 (56 – 59)	6,675	58 (56 – 59)
Male - %	14	6,675	73 (62 – 82)	8,381	73 (63 – 81)
Cardiac arrest data					
OHCA - %	14	6,675	89	8,381	89
Acute coronary syndrome - %	1	60	43 (31 – 56)	60	35 (24 – 48)
Pulmonary embolism - %	1	60	5 (2 – 14)	60	3 (0 – 12)
Shockable rhythm - %	14	6,675	50 (39 – 61)	6,675	43 (31 – 56)
Low-flow time – minutes*	9	6,675	49 (39 – 58)	6,675	49 (39 – 58)
Target temperature control - %	7	5,332	23 (8 – 52)	5,332	13 (4 – 32)
Last CPC 1 or 2 reported	12	2,315	12 (7 – 20)	2,315	9 (7 – 12)
Last survival reported - %	14	6,675	23 (15 – 33)	6,675	17 (10 – 26)

ECPR - extracorporeal cardiopulmonary resuscitation; CPR - cardiopulmonary resuscitation; 95%CI - 95% confidence interval; OHCA - out-of-hospital cardiac arrest; CPC - cerebral performance category. * The time until extracorporeal membrane oxygenation blood flow or return of spontaneous circulation.

Table 5S - Pooled characteristics of patients and cardiac arrests of the six logistic regression adjusted studies

Characteristic	ECPR			Conventional CPR	
	Number of studies	Sample size	Pooled mean/ incidence (95%CI)	Sample size	Pooled mean/ incidence (95%CI)
Age (years)	6	1,061	56 (53 – 59)	14,894	56 (53 – 59)
Male - %	6	1,061	84 (76 – 89)	14,894	68 (59 – 79)
Cardiac arrest data					
OHCA - %	6	1,061	100	14,894	100
Acute coronary syndrome - %	4	876	57 (42 – 71)	1,071	45 (36 – 53)
Pulmonary embolism - %	2	101	2 (0 – 7)	914	1 (0 – 2)
Shockable rhythm - %	5	1,041	57 (47 – 66)	2,188	35 (16 – 60)
Low-flow time – minutes *	5	1,041	44 (29 – 58)	2,188	44 (29 – 58)
Target temperature control - %	3	795	81 (2 – 100)	12,863	92 (0 – 99)
Last CPC 1 or 2 reported	3	430	24 (9 – 48)	1,171	6 (1 – 22)
Last survival reported - %	5	901	20 (11 – 34)	13,920	8 (2 – 25)

ECPR - extracorporeal cardiopulmonary resuscitation; CPR - cardiopulmonary resuscitation; 95%CI - 95% confidence interval; OHCA - out-of-hospital cardiac arres; CPC - cerebral performance category. * The time until extracorporeal membrane oxygenation blood flow or return of spontaneous circulation.

Table 6S - Pooled characteristics of patients and cardiac arrests of the eight non-matched and non-adjusted studies

Characteristic	ECPR			Conventional CPR	
	Number of studies	Sample size	Pooled mean/ incidence (95%CI)	Sample size	Pooled mean/ incidence (95%CI)
Age (years)	8	6,143	58 (56 – 60)	213,540	58 (56 – 60)
Male - %	8	6,143	82 (76 – 87)	213,540	74 (66 – 82)
Cardiac arrest data					
OHCA - %	8	6,143	75	213,540	75
Acute coronary syndrome - %	3	5,693	68 (8 – 98)	710	57 (3 – 98)
Pulmonary embolism - %	2	5,650	12 (2 – 46)	493	4 (3 – 6)
Shockable rhythm - %	8	6,143	55 (26 – 81)	213,540	26 (2 – 85)
Low-flow time – minutes *	8	6,143	33 (2 – 64)	213,540	33 (2 – 64)
Target temperature control - %	3	5,902	71 (14 – 97)	212,335	51 (0 – 100)
Last CPC 1 or 2 reported	7	6,100	20 (13 – 28)	213,517	7 (2 – 21)
Last survival reported - %	8	6,143	27 (20 – 35)	213,540	11 (4 – 26)

ECPR - extracorporeal cardiopulmonary resuscitation; CPR - cardiopulmonary resuscitation; 95%CI - 95% confidence interval; OHCA - out-of-hospital cardiac arres; CPC - cerebral performance category. * The time until extracorporeal membrane oxygenation blood flow or return of spontaneous circulation.

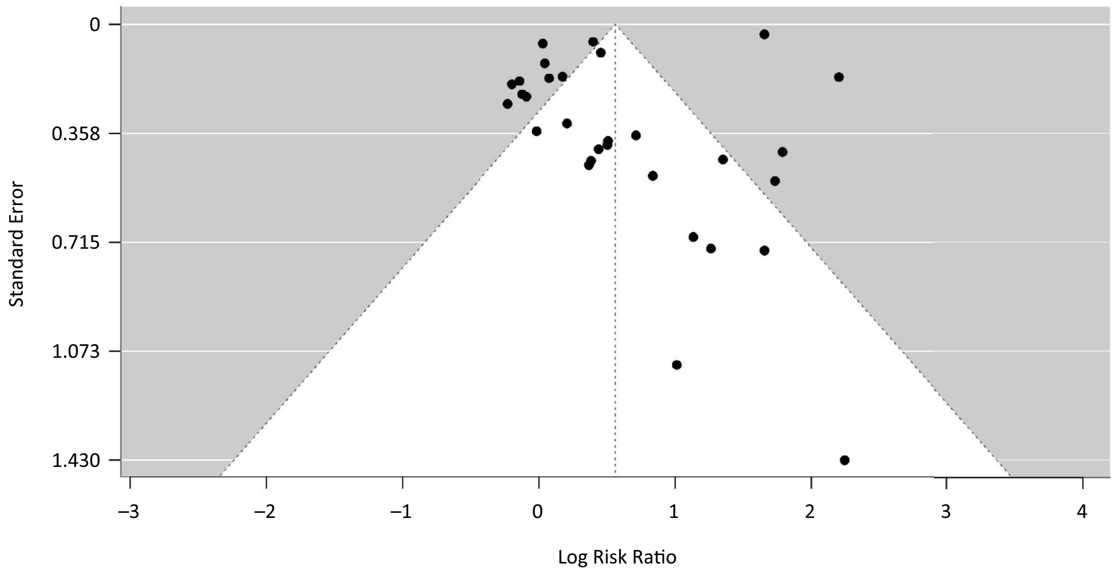

Figure 4S - Funnel plot of all included studies showing the publication bias.

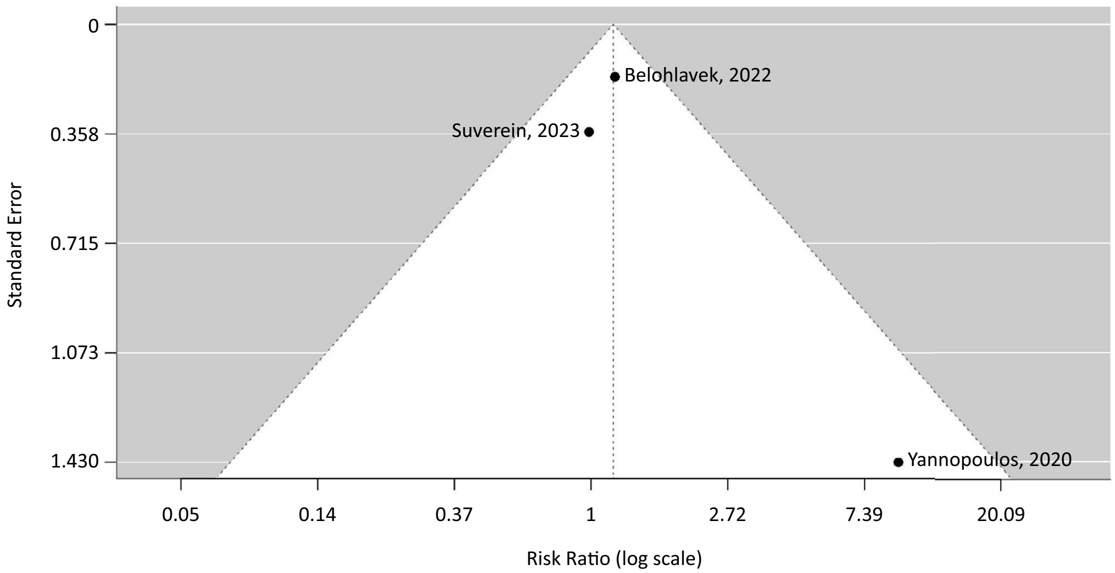

Figure 5S - Funnel plot of randomized controlled studies evaluated showing the publication bias.

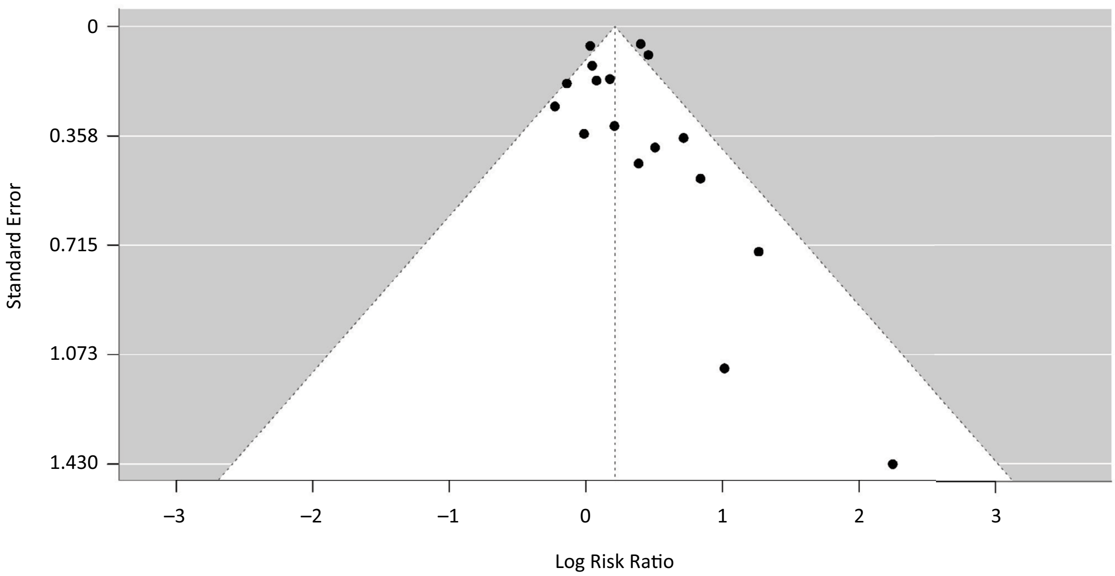

Figure 6S - Funnel plot of randomized, propensity score matched and coarsened exact matched studies evaluated showing the publication bias.

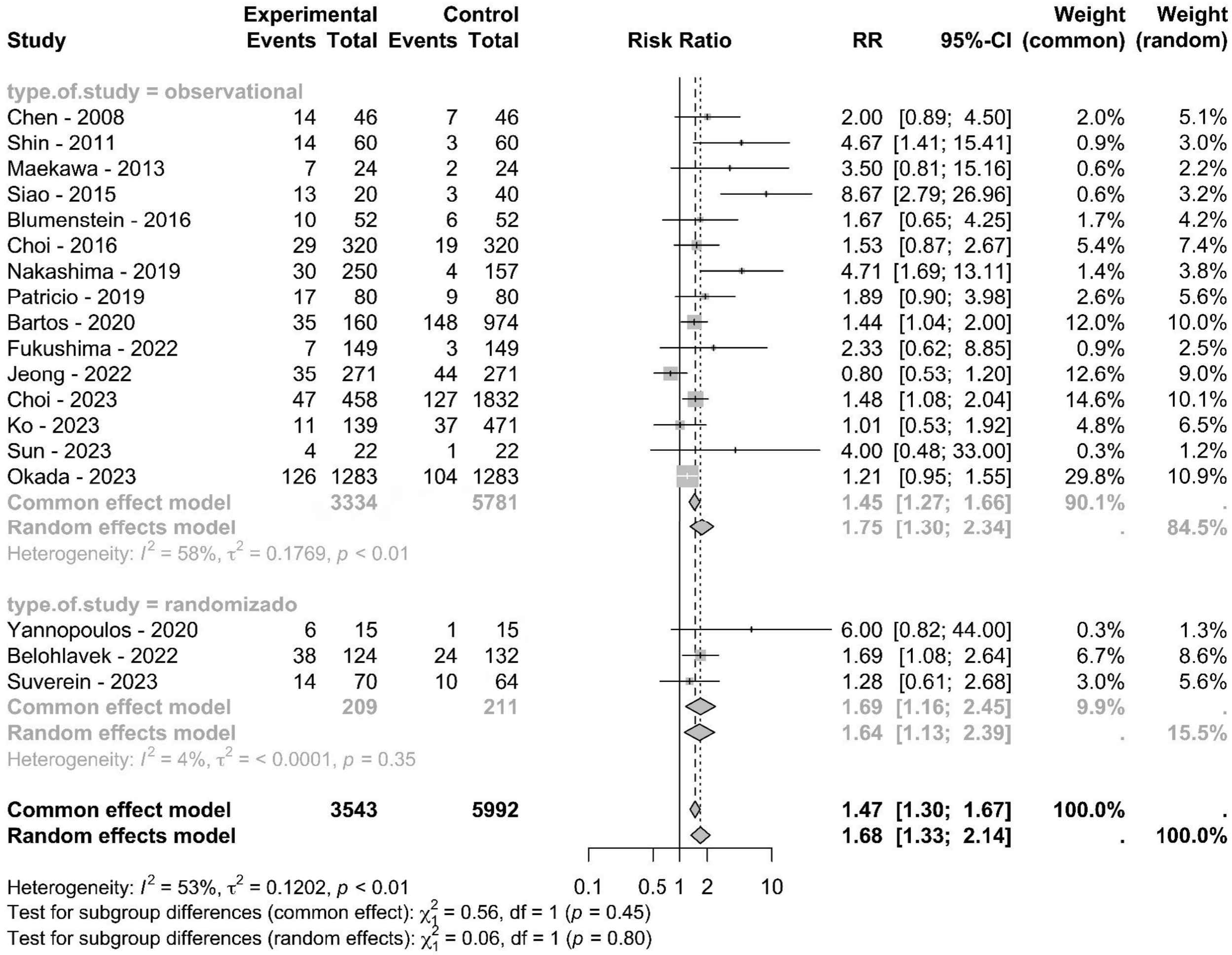

Figure 7S - Pooled results of randomized, propensity matched, coarsened exact matched, and adjusted with logistic regression studies, accomplishing the number of patients with the last reported cerebral performance category 1 or 2. The logistic regression adjusted studies had the number of deaths adjusted according to the odds ratio and normalizing the control group.

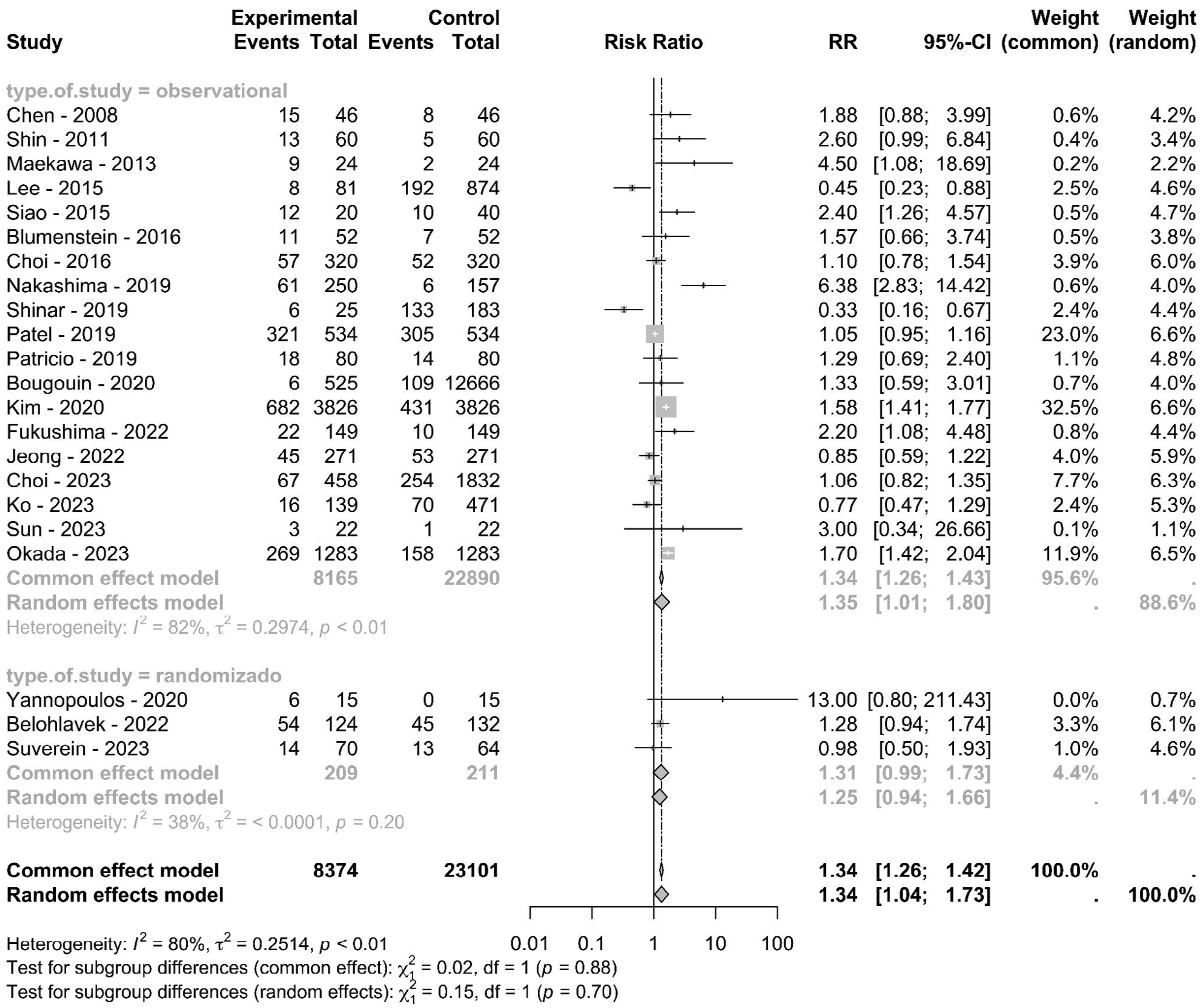

Figure 8S - Pooled results of randomized, propensity matched, coarsened exact matched, and adjusted with logistic regression studies, accomplishing the number of patients with the last reported survival. The logistic regression adjusted studies had the number of deaths adjusted according to the odds ratio and normalizing the control group.

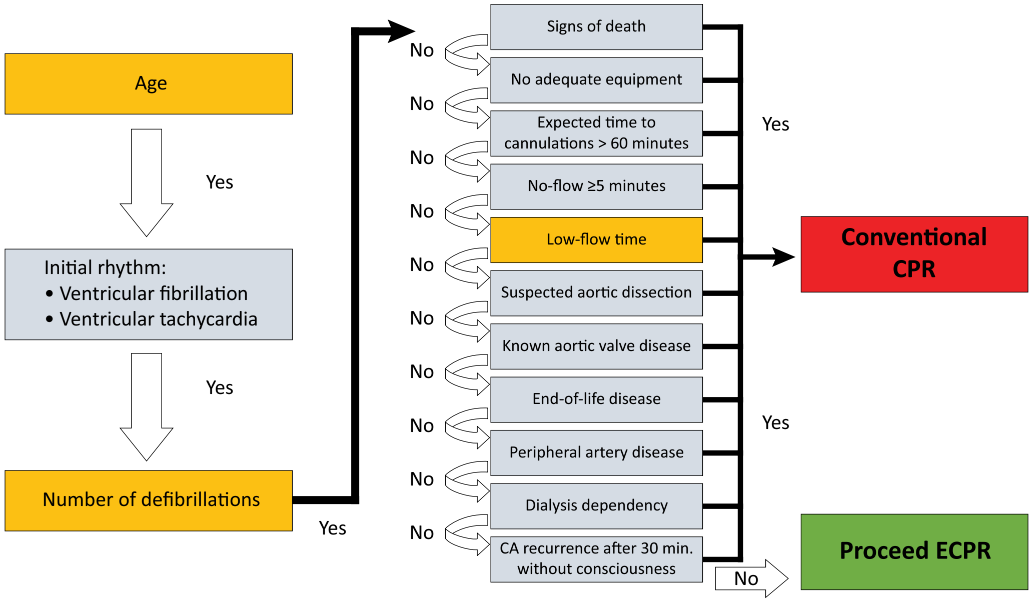

Figure 9S - Initial mindset to enrollment of patients to extracorporeal cardiopulmonary resuscitation. The tested variables are with orange background color.

CCPR - conventional cardiopulmonary resuscitation; ECPR - extracorporeal cardiopulmonary resuscitation.

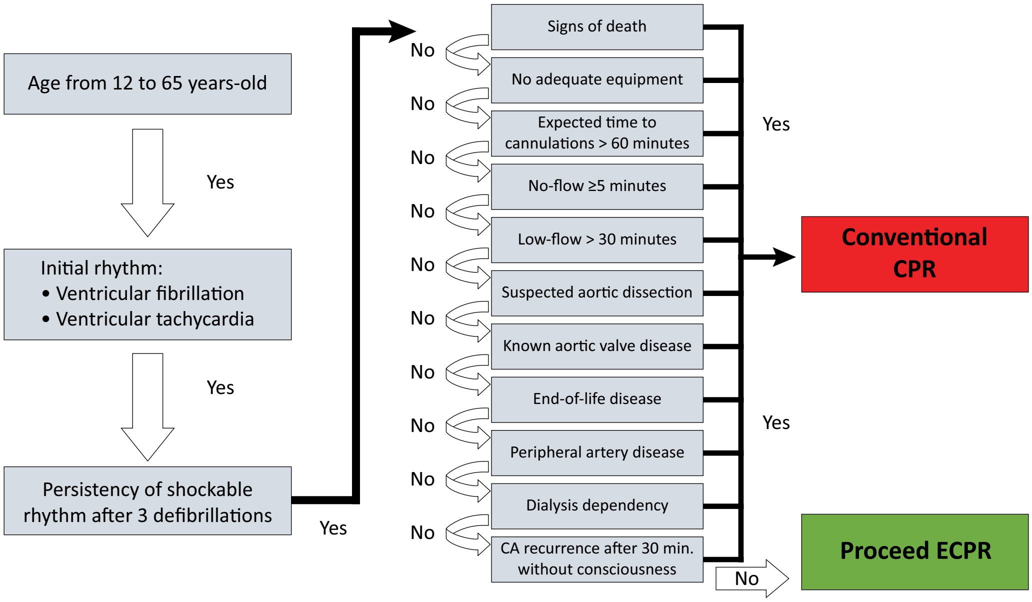

Figure 10S - Final criteria for enrollment of patients at the initial phase of our extracorporeal cardiopulmonary resuscitation program.

CCPR - conventional cardiopulmonary resuscitation; ECPR - extracorporeal cardiopulmonary resuscitation.

REFERENCES

1. Chen YS, Lin JW, Yu HY, Ko WJ, Jerng JS, Chang WT, et al. Cardiopulmonary resuscitation with assisted extracorporeal life-support versus conventional cardiopulmonary resuscitation in adults with in-hospital cardiac arrest: an observational study and propensity analysis. Lancet. 2008;372(9638):554-61.
2. Shin TG, Choi JH, Jo IJ, Sim MS, Song HG, Jeong YK, et al. Extracorporeal cardiopulmonary resuscitation in patients with inhospital cardiac arrest: A comparison with conventional cardiopulmonary resuscitation. Crit Care Med. 2011;39(1):1-7.
3. Maekawa K, Tanno K, Hase M, Mori K, Asai Y. Extracorporeal cardiopulmonary resuscitation for patients with out-of-hospital cardiac arrest of cardiac origin: a propensity-matched study and predictor analysis. Crit Care Med. 2013;41(5):1186-96.
4. Chou TH, Fang CC, Yen ZS, Lee CC, Chen YS, Ko WJ, et al. An observational study of extracorporeal CPR for in-hospital cardiac arrest secondary to myocardial infarction. Emerg Med J. 2014;31(6):441-7.
5. Sakamoto T, Morimura N, Nagao K, Asai Y, Yokota H, Nara S, et al.; SAVE-J Study Group. Extracorporeal cardiopulmonary resuscitation versus conventional cardiopulmonary resuscitation in adults with out-of-hospital cardiac arrest: a prospective observational study. Resuscitation. 2014;85(6):762-8.
6. Lee SH, Jung JS, Lee KH, Kim HJ, Son HS, Sun K. Comparison of extracorporeal cardiopulmonary resuscitation with conventional cardiopulmonary resuscitation: is extracorporeal cardiopulmonary resuscitation beneficial? Korean J Thorac Cardiovasc Surg. 2015;48(5):318-27.
7. Siao FY, Chiu CC, Chiu CW, Chen YC, Chen YL, Hsieh YK, et al. Managing cardiac arrest with refractory ventricular fibrillation in the emergency department: conventional cardiopulmonary resuscitation versus extracorporeal cardiopulmonary resuscitation. Resuscitation. 2015;92:70-6.
8. Blumenstein J, Leick J, Liebetrau C, Kempfert J, Gaede L, Groß S, et al. Extracorporeal life support in cardiovascular patients with observed refractory in-hospital cardiac arrest is associated with favourable short and long-term outcomes: A propensity-matched analysis. Eur Heart J Acute Cardiovasc Care. 2016;5(7):13-22.
9. Choi DH, Kim YJ, Ryoo SM, Sohn CH, Ahn S, Seo DW, et al. Extracorporeal cardiopulmonary resuscitation among patients with out-of-hospital cardiac arrest. Clin Exp Emerg Med. 2016;3(3):132-8.
10. Choi DS, Kim T, Ro YS, Ahn KO, Lee EJ, Hwang SS, et al. Extracorporeal life support and survival after out-of-hospital cardiac arrest in a nationwide registry: A propensity score-matched analysis. Resuscitation. 2016;99:26-32.
11. Nakashima T, Noguchi T, Tahara Y, Nishimura K, Ogata S, Yasuda S, et al.; SAVE-J Group. Patients with refractory out-of-cardiac arrest and sustained ventricular fibrillation as candidates for extracorporeal cardiopulmonary resuscitation: prospective multi-center observational study. Circ J. 2019;83(5):1011-8.
12. Shinar Z, Plantmason L, Reynolds J, Dembitsky W, Bellezzo J, Ho C, et al. Emergency physician-initiated resuscitative extracorporeal membrane oxygenation. J Emerg Med. 2019;56(6):666-73.
13. Patel JK, Meng H, Qadeer A, Parikh PB. Impact of extracorporeal membrane oxygenation on mortality in adults with cardiac arrest. Am J Cardiol. 2019;124(12):1857-61.
14. Patricio D, Peluso L, Brasseur A, Lheureux O, Belliato M, Vincent JL, et al. Comparison of extracorporeal and conventional cardiopulmonary resuscitation: a retrospective propensity score matched study. Crit Care. 2019;23(1):27.
15. Bartos JA, Grunau B, Carlson C, Duval S, Ripeckyj A, Kalra R, et al. Improved survival with extracorporeal cardiopulmonary resuscitation despite progressive metabolic derangement associated with prolonged resuscitation. Circulation. 2020;141(11):877-86.
16. Bougouin W, Dumas F, Lamhaut L, Marijon E, Carli P, Combes A, et al.; Sudden Death Expertise Center investigators. Extracorporeal cardiopulmonary resuscitation in out-of-hospital cardiac arrest: a registry study. Eur Heart J. 2020;41(21):1961-71.
17. Kim SJ, Han KS, Lee EJ, Lee SJ, Lee JS, Lee SW. Association between extracorporeal membrane oxygenation (Ecmo) and mortality in the patients with cardiac arrest: A nation-wide population-based study with propensity score matched analysis. J Clin Med. 2020;9(11):1-16.
18. Nakajima M, H Kaszynski R, Goto H, Matsui H, Fushimi K, Yamaguchi Y, et al. Current trends and outcomes of extracorporeal cardiopulmonary resuscitation for out-of-hospital cardiac arrest in Japan: a nationwide observational study. Resusc Plus. 2020;4:100048.
19. Shin YS, Kim YJ, Ryoo SM, Sohn CH, Ahn S, Seo DW, et al. Promising candidates for extracorporeal cardiopulmonary resuscitation for out-of-hospital cardiac arrest. Sci Rep. 2020;10(1):22180.
20. Yannopoulos D, Bartos J, Raveendran G, Walser E, Connett J, Murray TA, et al. Advanced reperfusion strategies for patients with out-of-hospital cardiac arrest and refractory ventricular fibrillation (ARREST): a phase 2, single centre, open-label, randomised controlled trial. Lancet. 2020;396(10265):1807-16.
21. Yoshida T, Fujitani S, Wakatake H, Kitano Y, Yoshida M, Tsutsumi K, et al. Exploratory observational study of extracorporeal cardiopulmonary resuscitation for nonshockable out-of-hospital cardiac arrest occurring after an emergency medical services arrival: SOS-KANTO 2012 Study Report. J Emerg Med. 2020;58(3):375-84.
22. Alm-Kruse K, Sørensen G, Osbakk SA, Sunde K, Bendz B, Andersen GØ, et al. Outcome in refractory out-of-hospital cardiac arrest before and after implementation of an ECPR protocol. Resuscitation. 2021;162:35-42.
23. Belohlavek J, Smalcova J, Rob D, Franek O, Smid O, Pokorna M, et al.; Prague OHCA Study Group. Effect of intra-arrest transport, extracorporeal cardiopulmonary resuscitation, and immediate invasive assessment and treatment on functional neurologic outcome in refractory out-of-hospital cardiac arrest: a randomized clinical trial. JAMA. 2022;327(8):737-47.
24. Ben-Hamouda N, Ltaief Z, Kirsch M, Novy J, Liaudet L, Oddo M, et al. Neuroprognostication under ECMO after cardiac arrest: are classical tools still performant? Neurocrit Care. 2022;37(1):293-301.
25. Fukushima K, Aoki M, Nakajima J, Aramaki Y, Ichikawa Y, Isshiki Y, et al. Favorable prognosis by extracorporeal cardiopulmonary resuscitation for subsequent shockable rhythm patients. Am J Emerg Med. 2022;53:144-9.
26. Jeong D, Lee GT, Park JE, Chang H, Kim T, Cha WC, et al. Extracorporeal life-support for out-of-hospital cardiac arrest: a nationwide multicenter study. Shock. 2022;57(5):680-6.
27. Choi Y, Park JH, Jeong J, Kim YJ, Song KJ, Shin SD. Extracorporeal cardiopulmonary resuscitation for adult out-of-hospital cardiac arrest patients: time-dependent propensity score-sequential matching analysis from a nationwide population-based registry. Crit Care. 2023;27(1):87.
28. Ko K, Kim YH, Lee JH, Lee KY, Hwang SY, Jin MH. The Effects of Extracorporeal Cardiopulmonary Resuscitation According to Covariate Adjustment. ASAIO J. 2023;69(2):191-7.
29. Suverein MM, Delnoij TS, Lorusso R, Brandon Bravo Bruinsma GJ, Otterspoor L, Elzo Kraemer CV, et al. Early extracorporeal CPR for refractory out-of-hospital cardiac arrest. N Engl J Med. 2023;388(4):299-309.
30. Sun P, Liu W, Li M, Zhang L, Liu LN, Liu ZX, et al. Extracorporeal cardiopulmonary resuscitation versus conventional cardiopulmonary resuscitation for patients with refractory out-of-hospital cardiac arrest: A retrospective propensity matching analysis. Perfusion. 2023;2676591231222365.
31. Okada Y, Komukai S, Irisawa T, Yamada T, Yoshiya K, Park C, et al. In-hospital extracorporeal cardiopulmonary resuscitation for patients with out-of-hospital cardiac arrest: an analysis by time-dependent propensity score matching using a nationwide database in Japan. Crit Care. 2023;27(1):442.
